# Profiles of immune cell infiltration and immune-related genes in the tumor microenvironment of osteosarcoma cancer

**DOI:** 10.1186/s12885-021-09042-6

**Published:** 2021-12-18

**Authors:** Ruixuan Liu, Yuhang Hu, Tianyi Liu, Yansong Wang

**Affiliations:** 1 Department of Spine Surgery, The First Affiliated Hospital of Harbin Medical University, Harbin, 150001 Heilongjiang Province China; 2 Department of Neurology, The First Affiliated Hospital of Harbin Medical University, Harbin, 150001 Heilongjiang Province China

**Keywords:** Osteosarcoma (OS), Immune cell populations, Immune cell infiltration, Survival analysis, Biomarker

## Abstract

**Backgrounds:**

Osteosarcomas are one of the most common primary malignant tumors of bone. It primarily occurs in children and adolescents, with the second highest incidence among people over 50 years old. Although there were immense improvements in the survival of patients with osteosarcoma in the past 30 years, targetable mutations and agents of osteosarcomas still have been generally not satisfactory. Therefore, it is of great importance to further explore the highly specialized immune environment of bone, genes related to macrophage infiltration and potential therapeutic biomarkers and targets.

**Methods:**

The 11 expression data sets of OS tissues and the 11 data sets of adjacent non-tumorous tissues available in the GEO database GSE126209 were used to conduct immune infiltration analysis. Then, through WGCNA analysis, we acquired the co-expression modules related to Mast cells activated and performed the GO and KEGG enrichment analysis. Next, we did the survival prognosis analysis and plotted a survival curve. Finally, we analyzed the COX multivariate regression of gene expression on clinical parameters and drew forest maps for visualization by the forest plot package.

**Results:**

OS disease-related immune cell populations, mainly Mast cells activated, have higher cell content (*p* = 0.006) than the normal group. Then, we identified co-expression modules related to Mast cells activated. In sum, a total of 822 genes from the top three strongest positive correlation module MEbrown4, MEdarkslateblue and MEnavajowhite2 and the strongest negative correlation module MEdarkturquoise. From that, we identified nine genes with different levels in immune cell infiltration related to osteosarcoma, eight of which including *SORBS2*, *BAIAP2L2, ATAD2, CYGB, PAMR1, PSIP1, SNAPC3* and *ZDHHC21* in their low abundance have higher disease-free survival probability than the group in their high abundances.

**Conclusion:**

These results could assist clinicians to select targets for immunotherapies and individualize treatment strategies for patients with OS.

**Supplementary Information:**

The online version contains supplementary material available at 10.1186/s12885-021-09042-6.

## Introduction

Osteosarcoma (OS), as the most common primary malignant tumor of bone, was frequently lethal via metastasis to the lung and primarily affected children and adolescents [1–10]. Moreover, there is a second peak in incidence in those over the age of 50. According to statistic, the incidence of OS worldwide was approximately one to three cases annually per million. Moreover, the 5-year survival rate for recurrent or metastatic OS is less than 25% [11, 12], though patients of OS can be typically treated with surgery and intensive adjuvant chemotherapy. Furthermore, the survival rate of patients with OS treated with surgery (mainstay of curative OS treatment) alone is approximately 15–17% [11, 13]. Therefore, there is an urgent requirement to develop effective and safe potential future therapies for patients with OS, which exerts minimal cytotoxicity on healthy tissue [14–25].

Osteosarcomas mostly occur in the long bones of the limbs covering the femur, the tibia and the humerus, and less commonly in the skull, the jaw or the pelvis with the distinct molecular and biological behaviors [26, 27]. Bone has a highly specialized immune environment and many immune signaling pathways are extremely important in bone homeostasis. The success of the innate immune stimulant mifamurtide in the adjuvant treatment of non-metastatic OS suggests that newer immune-based treatments, such as immune checkpoint inhibitors, may substantially improve treatment effect [28, 29]. The immune environment of OS is mainly composed by T-lymphocytes, macrophages and other subpopulations including B-lymphocytes and mast cells. OS cells control the recruitment and differentiation of immune infiltrating cells, establish a local immune tolerance environment conducive to tumor growth, drug resistance and metastasis and affect the balance between M1 and M2 macrophage subtypes.

In summary, given the high incidence and mortality, the detection, risk assessment and survival prognostic analyses are essential for improving the diagnosis and treatment of OS [1, 30]. Accordingly, it has a great necessity and urgency of searching for novel diagnosis and treatment targets as well as prognosis biomarkers, especially the high relationship with the markers of a subpopulation of Mast cells activated. Moreover, the identified candidate genes *SORBS2*, *BAIAP2L2* and others might be contributed to the improvement of the survival rate for OS cancer patients.

## Materials and methods

### Data mining of GEO database

Normalized sequencing data sets of Osteosarcoma (OS) downloaded from Gene Expression Omnibus (GEO, http://www.ncbi.nlm.nih.gov/geo/) GSE126209, which are mRNA expression profilings by high throughput sequencing of Illumina HiSeq 4000. GSE126209 from GPL20301 (*Homo sapiens*) comprises a total of 22 samples, 11 of which are from osteosarcoma tissues and 11 of which are fromthe adjacent normal tissues as controls.

### Immune infiltration analysis

According to the gene expression signature of specific cells, CIBERSORT [31] can estimate the content of specific cells in the expression profile of mixed cells by the deconvolution algorithm. Through the characteristic expression profiles of 22 immune cells provided by the CIBERSORT official website, we estimated the content of these 22 immune cells in the samples and visualized by the box plots based on the GSE126209 expression profile. We also visualized the relative proportions of the 22 immune cells by bar graphs. In addition, according to the content of 22 kinds of immune cells, the samples and immune cells are clustered and visualized with heat maps.

### Co-expression network analysis

WGCNA (weighted gene co-expression network analysis, weighted gene co-expression network analysis) is a method of the gene expression pattern analysis of multiple samples. It can cluster genes with similar expression patterns, and analyze the relationship between the modules and specific traits or phenotypes. Specially, according to the correlation between the expression levels of genes, a tree was constructed by clustering and the modules were divided. If certain genes always have similar expression trends in a physiological process or in different tissues, then these genes may be functionally related, and they can be defined as a module. Each gene module corresponds to a different color. Based on the correlation between traits (Mast cells infiltration score) and modules, the module with the highest correlation is selected for subsequent analysis. Here, the R package WGCNA [32] is used for co-expression analysis, and functional enrichment analysis is performed on the module genes with the highest phenotype correlation.

### Gene function enrichment analysis

Based on the database of gene ontology [33] and the KEGG pathway database with biochemical pathways [34], the candidate genes conducted the functional enrichment analysis. The statistical algorithm (Fisher’s exact test) was used to find out which specific functional items were most related to a group of genes. Each item in the analysis results corresponds to a statistical value *p*-value to represent significance. The smaller the *p*-value, the greater the relationship between the item and the input genes, that is, most of the genes in the group have the function described by the term.

### Survival prognosis analysis

Taking the median of gene expression as the cutoff of grouping, the samples were divided into two groups of high expression and low expression. Survival [35] packages were performed to analyze the survival difference between the two groups of samples by a survival curve. At the same time, we performed COX multivariate regression of gene expression on clinical parameters such as pathological staging, and drew forest maps for visualization by the forest plot package (Supplemental Table 1).

## Results

### Identification of differential immune cell populations

**Fig. 1:**
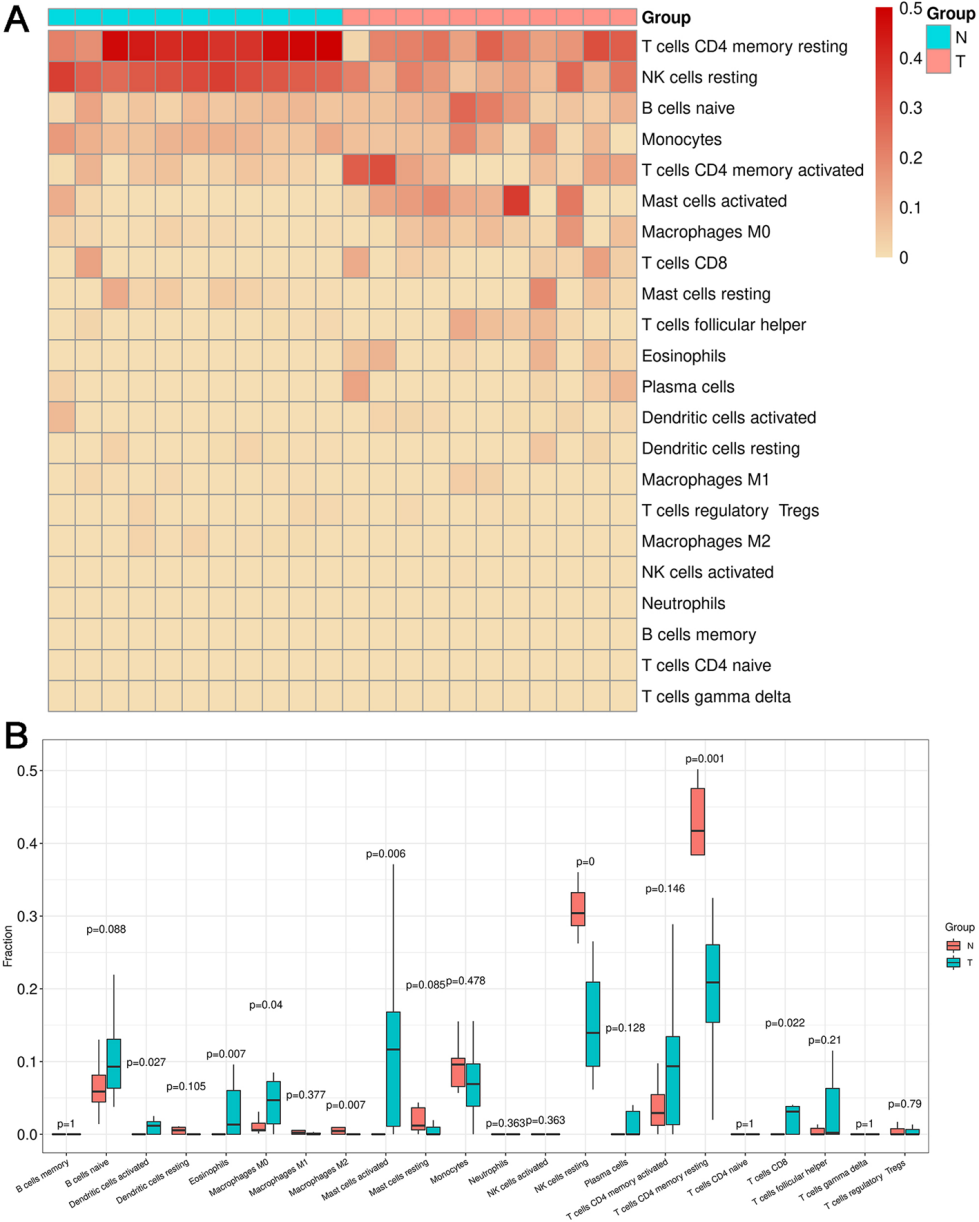
The analysis of differential immune cell populations. **A** Heat map of 22 immune cell scores of each sample (T means tumor tissue, N means normal tissue). **B** Box plot of the content of each immune cell in different groups

The 11 expression data sets of OS tissues and the 11 data sets of normal tissues were available for the analysis of differential immune cell populations. According to the expression level of each gene in the data set GSE126209, CIBERSORT was used to perform immune scores on the samples of the groups tumor and normal (Table 1 and Supplemental Table 2). The immune cell populations such as T cells CD4 memory resting and NK cell resting have higher scores in normal group, and T cells CD4 memory activated and Mast cells activated have higher scores in tumor cells related to OS (Fig. 1A). Moreover, T cells CD4 memory resting (*p* = 0.001) and NK cells resting (*p* = 0) also have higher cell content in normal group, and the immune cell population of T cells CD4 memory activated has higher cell content in the OS group (Fig. 1B). In addition, Mast cells activated have higher cell content (*p* = 0.006) in the OS group. The immune cell populations including dendritic cells activated, eosinophils, Macrophages M0 and T cells CD8 have higher cell content as well. At last, the immune cell population of Mast cells activated with higher scores in tumor cells related to OS and higher cell content with a significant *p* < 0.05 is selected for subsequent analysis.

**Table 1:** The score table of some immune cells in some samples

Sample	B cells naive	Mast cells activated	B cells memory
P14Normal	2.78E-02	0.00E+ 00	0
P14Tumor	2.67E-01	1.16E-01	0
P16Normal	8.00E-02	0.00E+ 00	0
P16Tumor	2.19E-01	1.01E-01	0
P20Normal	5.41E-02	0.00E+ 00	0

### Identification of infiltrating immune cells related genes based on co-expression modules

**Fig. 2:**
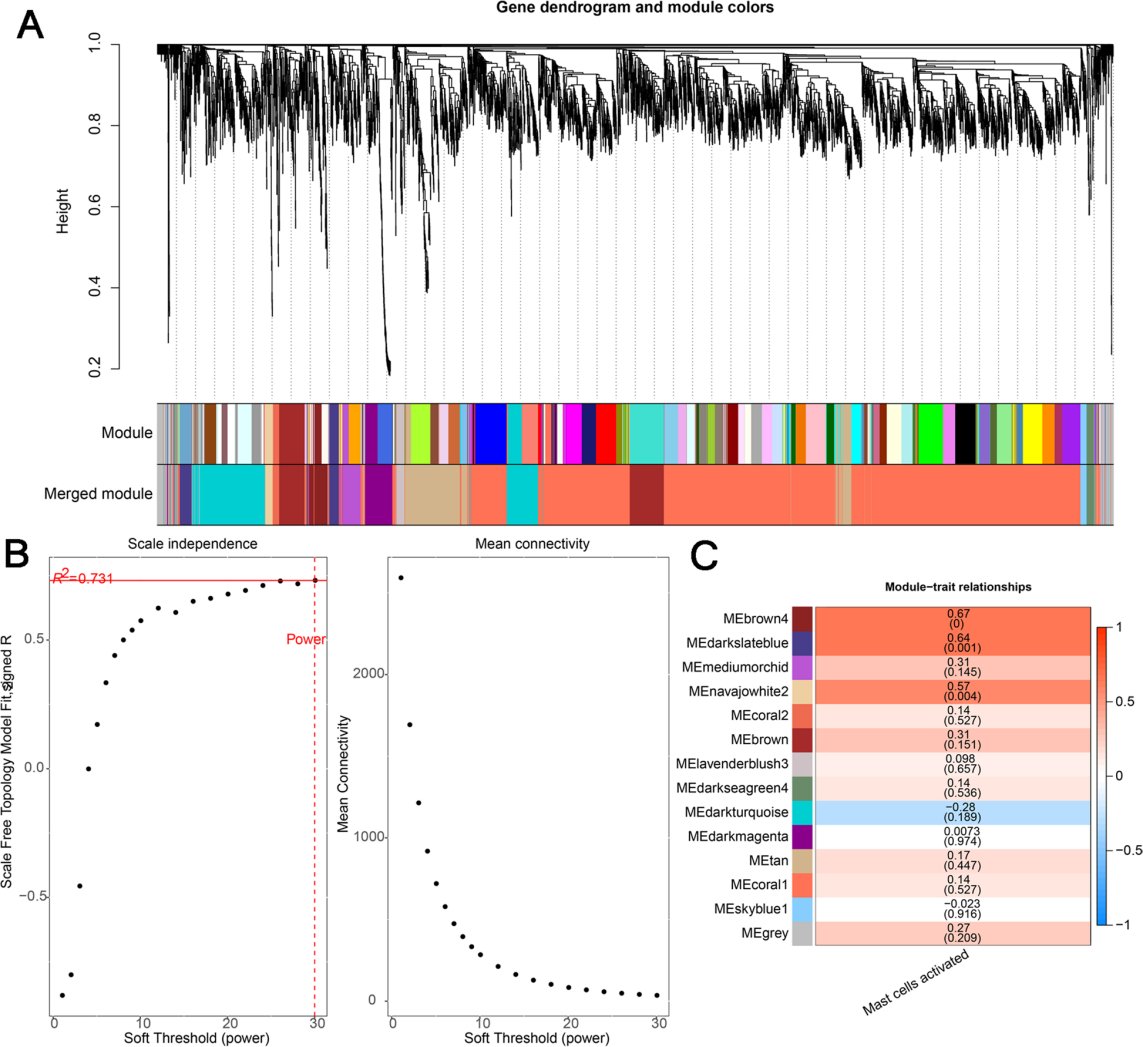
The analysis of co-expression modules. **A** The diagram of co-expression modules and the merged modules. **B** The diagram of co-expression power selection. **C** Heat map of the correlation between each module and Mast cells activated

With the correlation threshold to r > 0.8, we performed the co-expression analysis on the standardized expression data set GSE126209 (Fig. 2A, B). Due to the acquirement of scale free topology modules, the minimum of the correlation value r is 0.731 and the correlation threshold was set to 0.8 (Fig. 2B). Then, we calculated the correlation between each co-expression module and the immune score of Mast cells activated (Fig. 2C). From the overview of the correlation among modules and the statistics of gene numbers in each module (Table 2), there are 14 co-expression modules correlated with Mast cells activated. Notably, the module MEbrown4 consisting of 106 genes, has the strongest positive correlation with Mast cells activated, whose correlation coefficient is 0.673 with the significant *p* value of 4.33E-04. At the same time, the module MEdarkslateblue consisting of 116 genes, has the second strongest positive correlation with Mast cells activated, whose correlation coefficient is 0.643 with the significant *p* value of 9.35E-04. Instead, the module MEdarkturquoise has strongest negative correlation with Mast cells activated among all the 14 modules, whose correlation coefficient is − 0.284 with the significant *p* value of 1.89E-01.

**Table 2:** Overview of the correlation among modules and the statistics of gene numbers in each module

Module	Mast cells activated(cor)	***P***_val	Genes
MEbrown4	0.673	4.33E-04	106
MEdarkslateblue	0.643	9.35E-04	116
MEnavajowhite2	0.574	4.19E-03	42
MEmediumorchid	0.314	1.45E-01	109
MEbrown	0.309	1.51E-01	312
MEgrey	0.272	2.09E-01	151
MEtan	0.167	4.47E-01	402
MEcoral1	0.139	5.27E-01	2908
MEcoral2	0.139	5.27E-01	35
MEdarkseagreen4	0.136	5.36E-01	38
MElavenderblush3	0.098	6.57E-01	40
MEdarkmagenta	0.007	9.74E-01	151
MEskyblue1	−0.023	9.16E-01	32
MEdarkturquoise	−0.284	1.89E-01	558

### The potential clinical characteristics of modules related to mast cells activated

**Fig. 3:**
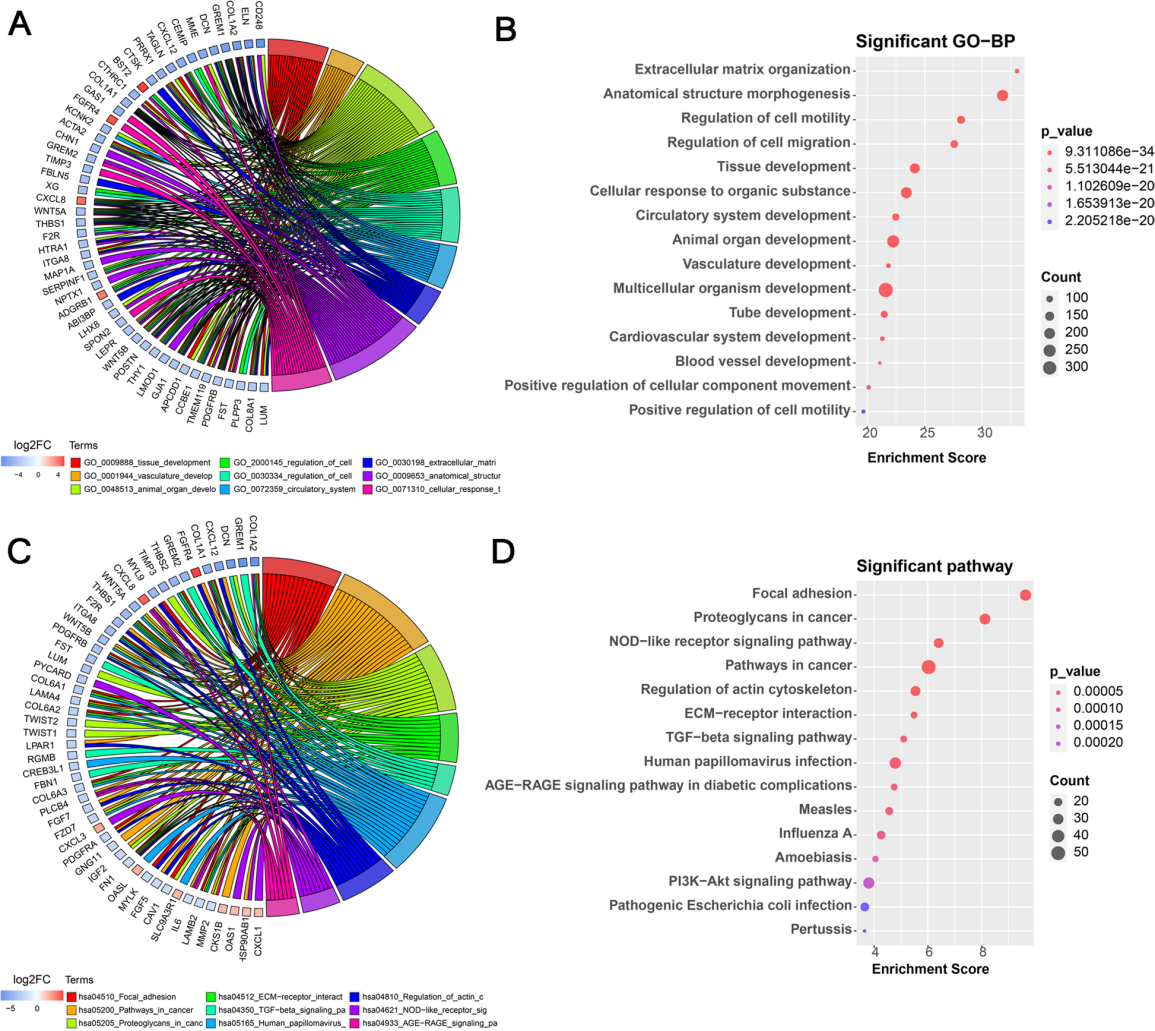
GO/KEGG function enrichment results. **A** Circle diagram of GO-BP gene enrichment results covering top 9 significant GO terms ranked in absolute log2FC values. **B** Bubble chart of the top 20 GO terms ranked in the enrichment score. **C** Circle diagram of KEGG gene enrichment results covering top 9 KEGG pathways ranked in absolute log2FC values. **D** Bubble chart of the top 20 KEGG significant pathways ranked in the enrichment score

**Fig. 4:**
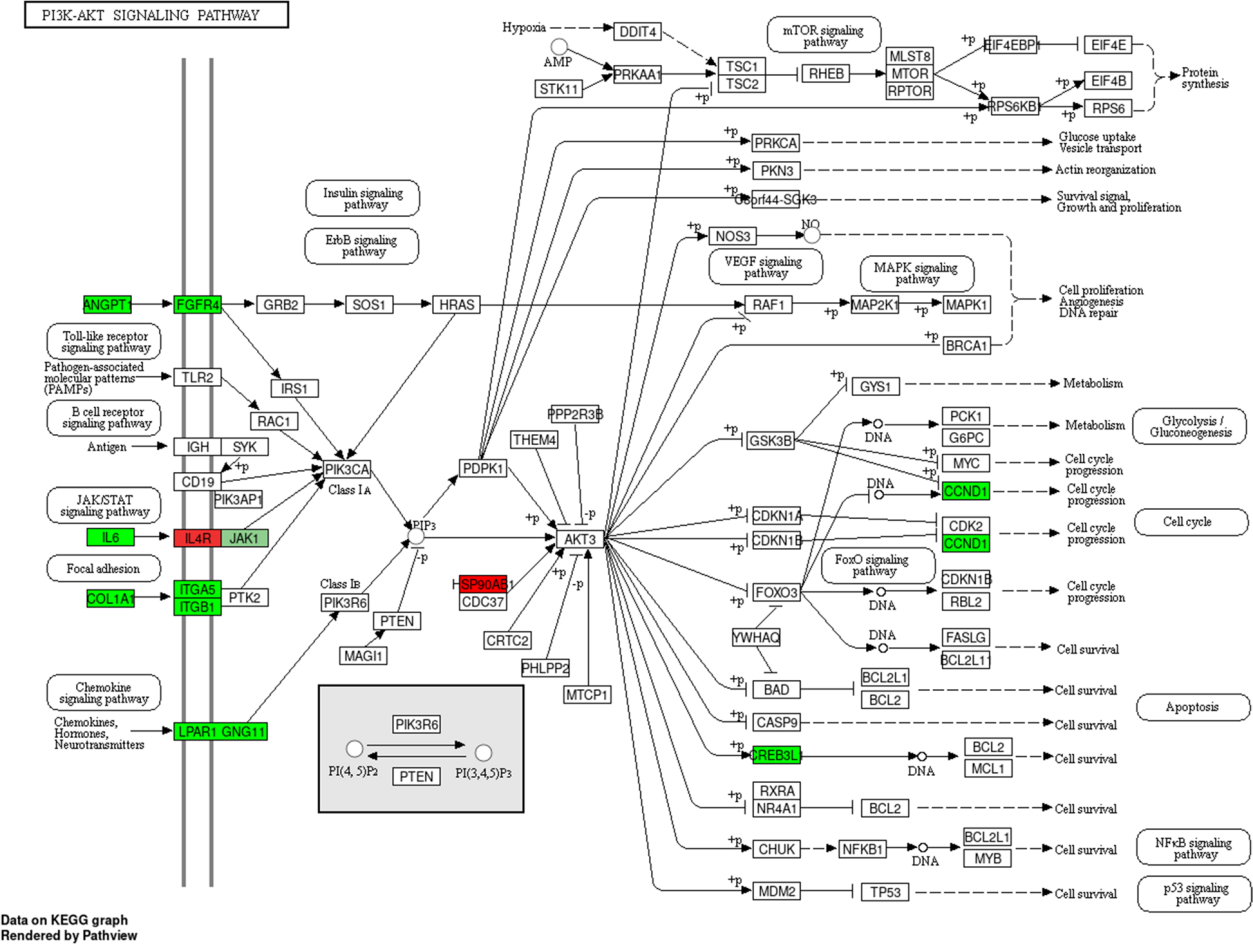
The location of the genes in the hsa04151 pathway

To further explore the potential clinical characteristics of modules related to Mast cells activated, a total of 822 genes from the top three strongest positive correlation module MEbrown4, MEdarkslateblue and MEnavajowhite2 and the strongest negative correlation module MEdarkturquoise were analyzed for GO/KEGG function enrichment (Table 3 and Fig. 3). Since there are many enrichment results, we selected the GO-BP enrichment results shown here, such as tissue development, multicellular organism development, anatomical structure morphogenesis, animal organ development, vasculature development and so on. For example, GO term “tissue development” contains genes such as *ACTA2, ALPK2, APCDD1, CCN1, CD109, CD81, CFL2, COL18A1, COL1A1, COL1A2, COL7A1, COL8A1, CRIP1, CSF1, CSRP1, CTHRC1* and so on. Of them, genes *COL1A2, CTHRC1, COL1A1, ACTA2, COL8A1*, and *APCDD1* were significantly down-regulated in the GO enriched results (Fig. 3A, B). As for KEGG pathways, the hsa04510 pathway focal adhesion was also enriched and includes the genes like *CAPN2, CAV1, CCND1, COL1A1, COL1A2, COL6A1, COL6A2, COL6A3, FN1, ITGA8, ITGB1, LAMA4, LAMB2, LAMC1, MAPK9, MYL9, MYLK, PDGFRA, PDGFRB, THBS1, THBS2*, and so forth (Fig. 3C, D). Similarly, the genes *LAMA4, ITGA8, COL1A1, COL1A2, PDGFRA, PDGFRB, THBS1, THBS2, MYLK, LAMB2* and *FN1* were significantly down-regulated in the KEGG enriched results. In addition, the hsa04151 pathway PI3K-AKT signaling pathway was enriched (Fig. 4). In this pathway, ANGPT1, FGFR4, IL6, COL1A1, ITGA5, ITGB1, LPAR1, JAK1, CREB3L and CCND1 showed down-regulation, while IL4R and HSP90AB1 showed up-regulation. All of them indicated that these genes from the key modules might participate in or be related to the pathways hsa04151, hsa04510, hsa05200, hsa05205, hsa04512, hsa04350, hsa05165, hsa04810 and other pathways (Fig. 3C, D).

**Table 3:** The partial enrichment results of GO-BP terms

ID	Term	***p***_value	Count	Enrichment_Score
GO:0007275	multicellular_organism_development	2.50E-22	341	21.603
GO:0007165	signal_transduction	2.23E-06	278	5.652
GO:0048513	animal_organ_development	5.63E-23	248	22.250
GO:0009653	anatomical_structure_morphogenesis	1.66E-32	212	31.779
GO:0019538	protein_metabolic_process	2.07E-02	212	1.684

### The hub genes for OS survival prognosis

**Fig. 5:**
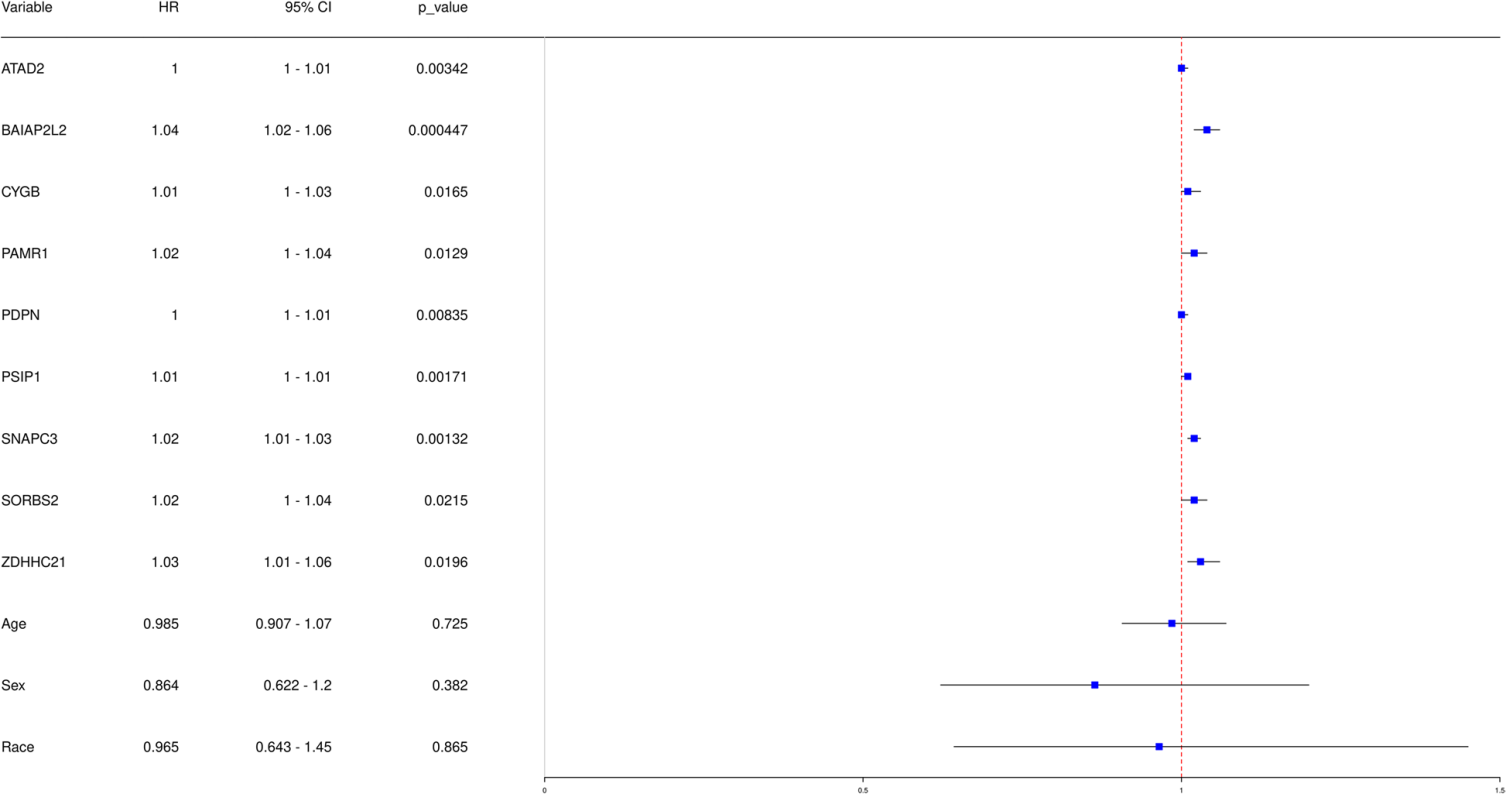
Forest plot of single factor regression results

According to the above positive and negative correlation modules got from the gene weight networks, we selected the 33, 4 and 9 genes with the weight of relationship pair > 0.3 from the positive correlation modules MEbrown4, MEdarkslateblue and MEnavajowhite2, respectively. In the meanwhile, we selected the 4 genes with the same cutoff from the negative correlation module MEdarkturquorise. In sum, a total of 50 hub genes were selected for single-factor cox regression pre-screening. Based on the overall survival cox regression *p* < 0.05, nine genes were selected for subsequent survival curve analysis and cox regression analysis (Table 4 and Fig. 5). The *p*-values of these nine hub genes were lower than the cutoff 0.05, while the *p*-values of the factors age, sex and race were higher than the cutoff (Fig. 5).

**Table 4:** Survival analysis gene pre-screen results

gene	os_cox_*p* value	gene	os_cox_***p*** value
BAIAP2L2	4.47E-04	PAMR1	1.29E-02
SNAPC3	1.32E-03	CYGB	1.65E-02
PSIP1	1.71E-03	ZDHHC21	1.96E-02
ATAD2	3.42E-03	SORBS2	2.15E-02
PDPN	8.35E-03

**Fig. 6:**
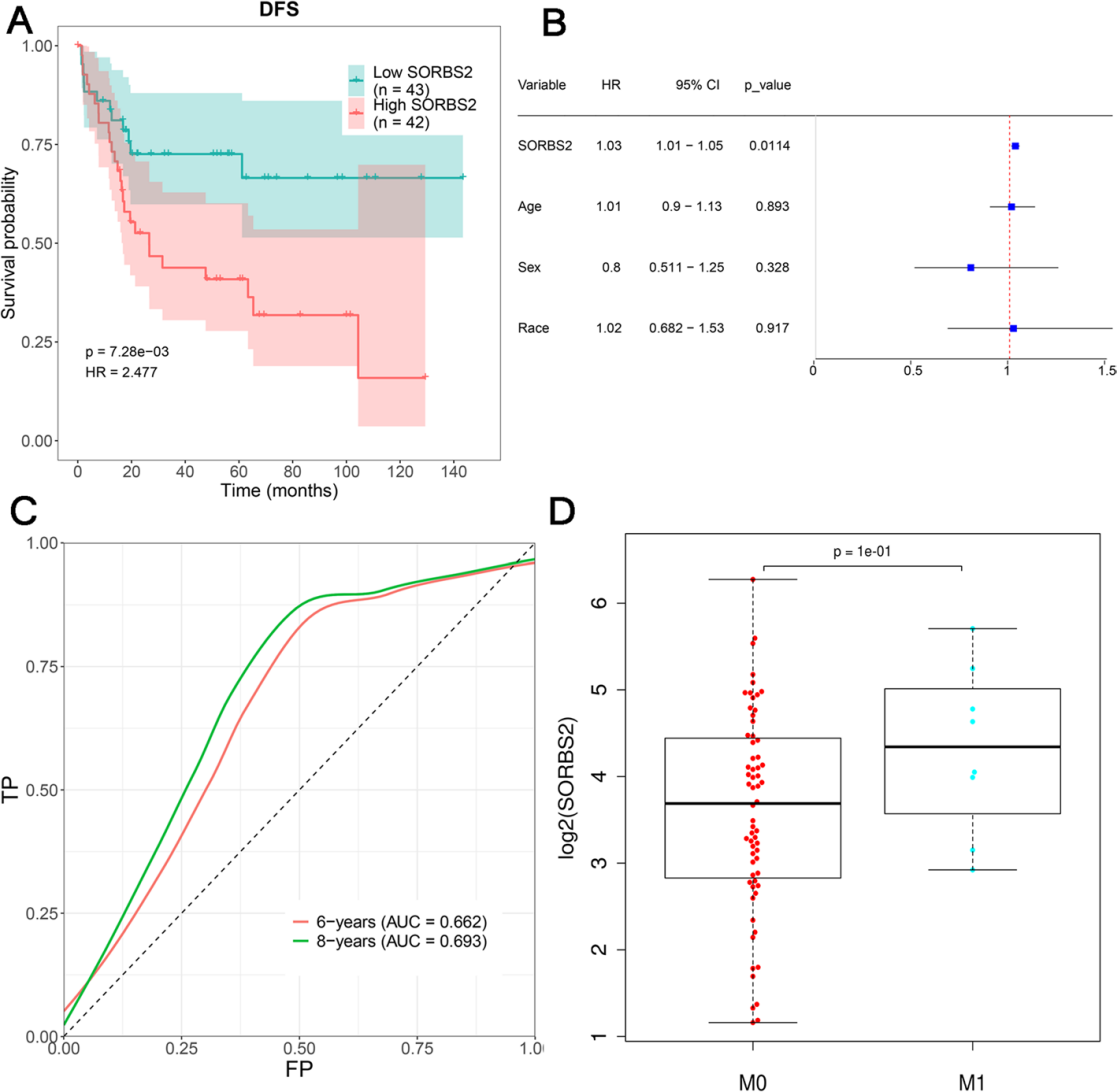
The survival and cox analysis of gene *SORBS2*. **A** Survival curve of gene *SORBS2*. **B** Multivariate regression forest plot of gene *SORBS2*. **C** ROC curve of cox model of gene *SORBS2*. **D** The expression of gene *SORBS2* in different tumor metastasis situations (M0 means non-metastatic, M1 means metastasis)

The nine hub genes were conducted the survival and cox analysis based on TARGET-OS sequencing data (Fig. 6). As shown in the result, the hazard ratio of *SORBS2* was 1.02 (95% CI 1 ~ 1.04, *p* value = 0.0215) in the forest plot of single factor regression results (Fig. 5). In addition, the group with low SORBS2 expression levels has higher disease-free survival probability than the group of high SORBS2 through the survival and cox analysis (Fig. 6). In the other hand, the group with lower abundances of BAIAP2L2, SNAPC3 and ZDHHC21 has higher disease-free survival probability than the group in their high abundances (Supplemental Fig. 1).

## Discussion

Osteosarcoma of bone is a tumor composed of malignant cells that produce osteoid [2, 13]. All of OS is highly malignant and about 80% of patients died with metastases, which remains challenges for treatment. Diagnosis, management and survival prognostic analyses, especially related to immune cell populations, are essential to improve the outcomes of treatment of OS [3, 4, 14–16, 26]. First of all, we identified the significant differential immune cell populations, which might be helpful for the prognosis and following research of OS. The immune cell population of T cells CD4 memory activated has higher cell content in the OS group, which might suggest that CD4+ T memory cells were activated and the number of these cells increased to participate in the destruction of OS tumor. The immune cell population of Mast cells activated with higher scores in OS cells and higher cell content with a significant *p* = 0.006 is selected for subsequent analysis. Although there are some other immune cell populations with higher scores in OS cells than those in normal cells like T cells CD4 memory activated, these populations are not significant. Furthermore, despite the immune cell populations including dendritic cells activated, eosinophils, Macrophages M0 and T cells CD8 have higher cell content as well, they do not have significantly higher scores in OS cells. Apparently, the mean value of the fraction in populations of Mast cells activated in group tumor is more than 0.1, whereas the mean values of the fraction in all the above other populations with higher cell content in OS group are less than 0.1.

Based on these, we further identified 14 co-expression modules correlated with Mast cells activated through the correlation between each co-expression module and the immune score of Mast cells activated. We conducted the GO and KEGG enrichment analysis of a total of 822 genes from the top three positive co-expression module and the unique negative co-expression module to explore their possible clinical characteristics. The GO analysis results indicated that the terms related to tissue development, multicellular organism development, anatomical structure morphogenesis, animal organ development and vasculature development were enriched. The term GO: 0009888 (tissue development) includes the down-regulated genes *TAGLN, APCDD1, TMEME119, LUM* and others. Furthermore, the enriched GO term GO: 0046649 (cellular response to organic substance) contains up-regulated gene FGFR4 and down-regulated genes *TIMP3, GAS1* and *SPON2*. There were pathways focal adhesion, proteoglycans in cancer, regulation of actin cytoskeleton, TGF-beta signaling pathway, pertussis and measles in KEGG enriched results covering genes *CXCL12, FGFR4, MYL9, F2R, LPAR1, FGF7* and so on. Moreover, the enriched PI3K-AKT pathway, as the most important oncogenic pathways in human cancer, also provided a revealing insight into the role of PI3K (phosphatidylinositol 3-kinase)-AKT signal in OS. These signals may contribute to one or more processes of tumorigenesis, proliferation, invasion in OS. In addition to nine genes related to differences in immune cell infiltration in osteosarcoma, the above-mentioned genes in GO or/and KEGG terms also might take effect on the biological process of OS.

All in all, Mast cells activated might be OS disease-related immune cell populations. Through the survival and cox analysis, we identified nine genes related to immune cell infiltration in osteosarcoma to help study novel potential diagnosis and candidate treatment targets as well as prognosis biomarkers.

## Conclusion

We brought forth the conclusion that disease-related immune cell populations, Mast cells activated, have higher cell content (*p* = 0.006) in the OS. Again, we identified nine genes related to differences in immune cell infiltration in osteosarcoma, four of which are *SORBS2, BAIAP2L2, SNAPC3* and *ZDHHC21* with low abundances and they have higher disease-free survival probability than the group with high abundances. These genes might be markers genes as the targets to help assist clinicians.

## Supplementary Information

**Additional file 1.**

**Additional file 2.**

**Additional file 3: Supplemental Figure 1.** The survival and cox analysis of gene *SORBS2*. **A.** Survival curve of gene *BAIAP2L2*. **B.** Multivariate regression forest plot of gene *BAIAP2L2*. **C.** ROC curve of cox model of gene *BAIAP2L2*. **D.** Survival curve of gene *SNAPC3*. **E.** Multivariate regression forest plot of gene *SNAPC3*. **F.** ROC curve of cox model of gene *SNAPC3*. **G.** Survival curve of gene *ZDHHC21*. **H.** Multivariate regression forest plot of gene *ZDHHC21*. **I.** ROC curve of cox model of gene *ZDHHC21*.

## Data Availability

The datasets generated and/or analyzed during the current study are available in the NCBI’s Gene Expression Omnibus repository, with the accession number of GSE126209.

## References

[1] Zhao J, Dean DC, Hornicek FJ, Yu X, Duan Z (2020). Emerging next-generation sequencing-based discoveries for targeted osteosarcoma therapy. Cancer Lett.

[2] Ritter J, Bielack SS (2010). Osteosarcoma. Ann Oncol.

[3] Dahlin DC. Pathology of Osteosarcoma. Clin Orthop Relat Res. 1975:23–32. 10.1097/00003086-197509000-00004. PMID: 168999. 10.1097/00003086-197509000-00004 168999

[4] Chen G, Xu Q, Zhang B, Dai M (2019). Parosteal osteosarcoma of the fibula in a middle-aged patient: a case report. Medicine.

[5] Abou Ghaida RR, Saoud RM, Bulbul M (2014). Primary osteosarcoma in a bladder diverticulum. Can J Urol.

[6] Marec-Berard P, Dalban C, Gaspar N (2020). A multicentric randomized phase II clinical trial evaluating high-dose thiotepa as adjuvant treatment to standard chemotherapy in patients with resectable relapsed osteosarcoma. Eur J Cancer (Oxford, England : 1990).

[7] Mathkour M, Garces J, Beard B, Bartholomew A, Sulaiman OA, Ware ML (2016). Primary high-grade Osteosarcoma of the Clivus: a case report and literature review. World Neurosurg.

[8] Ding Q, Zhang W, Cheng C, et al. Dioscin inhibits the growth of human osteosarcoma by inducing G2/M-phase arrest, apoptosis, and GSDME-dependent cell death in vitro and in vivo. J Cell Physiol. 2020;235:2911–24. 10.1002/jcp.29197. PMID: 31535374. 10.1002/jcp.29197 31535374

[9] Zhong J, Si L, Geng J (2020). Chondromyxoid fibroma-like osteosarcoma: a case series and literature review. BMC Musculoskelet Disord.

[10] Poon AC, Matsuyama A, Mutsaers AJ (2020). Recent and current clinical trials in canine appendicular osteosarcoma. Can Vet J.

[11] Link MP, Goorin AM, Miser AW (1986). The effect of adjuvant chemotherapy on relapse-free survival in patients with osteosarcoma of the extremity. N Engl J Med.

[12] Meyers PA, Healey JH, Chou AJ (2011). Addition of pamidronate to chemotherapy for the treatment of osteosarcoma. Cancer.

[13] Bernthal NM, Federman N, Eilber FR (2012). Long-term results (>25 years) of a randomized, prospective clinical trial evaluating chemotherapy in patients with high-grade, operable osteosarcoma. Cancer.

[14] Kansara M, Teng MW, Smyth MJ, Thomas DM (2014). Translational biology of osteosarcoma. Nat Rev Cancer.

[15] Kager L, Tamamyan G, Bielack S (2017). Novel insights and therapeutic interventions for pediatric osteosarcoma. Future Oncol (London, England).

[16] ElKordy MA, ElBaradie TS, ElSebai HI, KhairAlla SM, Amin AAE (2018). Osteosarcoma of the jaw: challenges in the diagnosis and treatment. J Egypt Natl Cancer Inst.

[17] Gold R, Oliveira F, Pool R (2019). Zygomatic arch Parosteal Osteosarcoma in dogs and a cat. Vet Pathol.

[18] Masrouha KZ, Khattab R, Tawil A (2012). A preliminary investigation of Beta-hCG expression in patients with osteosarcoma. The journal of bone and joint surgery. British.

[19] Mirabello L, Zhu B, Koster R (2020). Frequency of pathogenic Germline variants in Cancer-susceptibility genes in patients with Osteosarcoma. JAMA Oncol.

[20] Zheng C, Tang F, Min L, Hornicek F, Duan Z, Tu C (2020). PTEN in osteosarcoma: recent advances and the therapeutic potential. Biochimica et biophysica acta. Rev Cancer.

[21] Tian Z, Niu X, Yao W (2020). Receptor tyrosine kinases in Osteosarcoma treatment: which is the key Target?. Front Oncol.

[22] Zhou L, Yang C, Zhang N, Zhang X, Zhao T, Yu J (2020). Silencing METTL3 inhibits the proliferation and invasion of osteosarcoma by regulating ATAD2. Biomed Pharmacother.

[23] Ekhtiari S, Chiba K, Popovic S (2020). First case of osteosarcoma in a dinosaur: a multimodal diagnosis. Lancet Oncol.

[24] Wu W, Jing D, Meng Z (2020). FGD1 promotes tumor progression and regulates tumor immune response in osteosarcoma via inhibiting PTEN activity. Theranostics.

[25] Wang XZ, Zhang SF, Yang ZH, Ye ZW, Liu J (2020). Punicalagin suppresses osteosarcoma growth and metastasis by regulating NF-κB signaling. J Biol Regul Homeost Agents.

[26] Simpson E, Brown HL (2018). Understanding osteosarcomas. JAAPA.

[27] Mendenhall WM, Fernandes R, Werning JW, Vaysberg M, Malyapa RS, Mendenhall NP (2011). Head and neck osteosarcoma. Am J Otolaryngol.

[28] Hansen MF, Seton M, Merchant A (2006). Osteosarcoma in Paget's disease of bone. J Bone Miner Res.

[29] Raimondi L, De Luca A, Gallo A (2020). Osteosarcoma cell-derived exosomes affect tumor microenvironment by specific packaging of microRNAs. Carcinogenesis.

[30] Li X, Huang Q, Wang S, Huang Z, Yu F, Lin J (2020). HER4 promotes the growth and metastasis of osteosarcoma via the PI3K/AKT pathway. Acta Biochim Biophys Sin.

[31] Newman AM, Liu CL, Green MR (2015). Robust enumeration of cell subsets from tissue expression profiles. Nat Methods.

[32] Langfelder P, Horvath S. WGCNA: an R package for weighted correlation network analysis. BMC Bioinformatics. 2008;9:559–71. 10.1186/1471-2105-9-559 PMC2631488 19114008

[33] Ashburner M, Ball CA, Blake JA (2000). Gene ontology: tool for the unification of biology. The Gene Ontology Consortium. Nat Genet.

[34] Kanehisa M, Goto S (2000). KEGG: Kyoto encyclopedia of genes and genomes. Nucleic Acids Res.

[35] Li JCA (2003). Modeling survival data: extending the cox model. Sociol Method Res.

