# Supplementary figures and images for: Profiles of immune cell infiltration and immune-related genes in the tumor microenvironment of osteosarcoma cancer
Source: BMC Cancer. 2021 Dec 18;21:1345. doi: 10.1186/s12885-021-09042-6 (PMC8684084; doi:10.1186/s12885-021-09042-6)

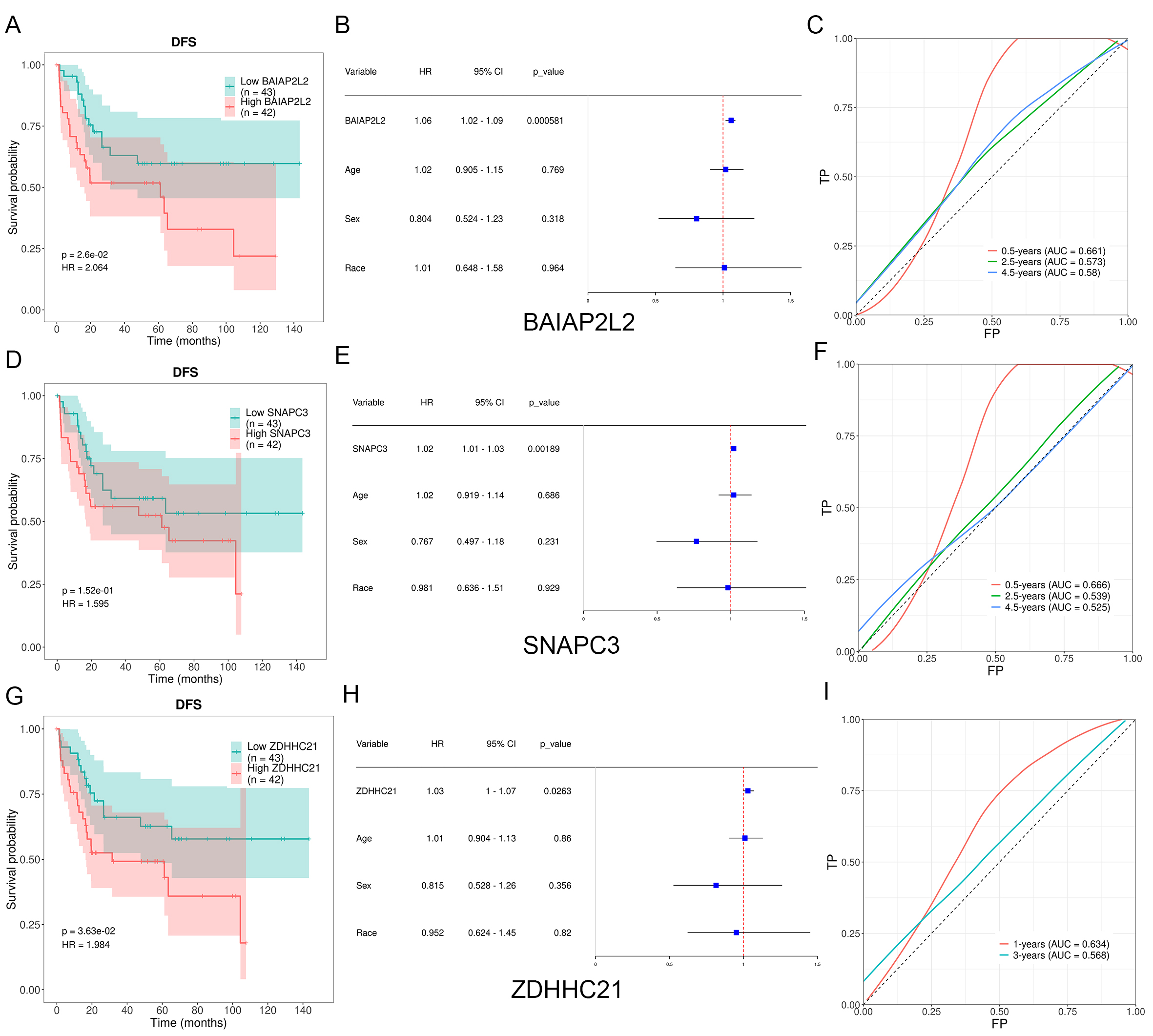

Supplement: Supplementary file 3 — Additional file 3: Supplemental Figure 1. The survival and cox analysis of gene SORBS2. A. Survival curve of gene BAIAP2L2. B. Multivariate regression forest plot of gene BAIAP2L2. C. ROC curve of cox model of gene BAIAP2L2. D. Survival curve of gene SNAPC3. E. Multivariate regression forest plot of gene SNAPC3. F. ROC curve of cox model of gene SNAPC3. G. Survival curve of gene ZDHHC21. H. Multivariate regression forest plot of gene ZDHHC21. I. ROC curve of cox model of gene ZDHHC21. [file 12885_2021_9042_MOESM3_ESM.jpg]
